# Polyphenols and Microbiota Modulation: Insights from Swine and Other Animal Models for Human Therapeutic Strategies

**DOI:** 10.3390/molecules29246026

**Published:** 2024-12-20

**Authors:** Andrei Cristian Anghel, Ionelia Țăranu, Alina Orțan, Simona Marcu Spinu, Mihaela Dragoi Cudalbeanu, Petronela Mihaela Rosu, Narcisa Elena Băbeanu

**Affiliations:** 1 Faculty of Biotechnologies, University of Agronomic Sciences and Veterinary Medicine of Bucharest, 59 Marasti Boulevard, 011464 Bucharest, Romania; andrei.anghel@ibna.ro (A.C.A.); narcisa.babeanu@usamv.ro (N.E.B.); 2 National Research-Development Institute for Animal Biology and Nutrition (IBNA), 1 Calea Bucuresti, 077015 Balotesti, Romania; ionelia.taranu@ibna.ro; 3 Faculty of Land Reclamation and Environmental Engineering, University of Agronomic Sciences and Veterinary Medicine of Bucharest, 59 MarastiBoulevard, 011464 Bucharest, Romania; simona.spinu@fifim.ro (S.M.S.); mcudalbeanu@gmail.com (M.D.C.); 4 Faculty of Veterinary Medicine, University of Agronomic Sciences and Veterinary Medicine of Bucharest, 59 Marasti Boulevard, 011464 Bucharest, Romania; petronela.rosu@fmvb.usamv.ro

**Keywords:** polyphenols, microbiota, gut, pig, mouse, rabbit, rat, human

## Abstract

High consumption of ultra-processed foods, rich in sugar and unhealthy fats, has been linked to the onset of numerous chronic diseases. Consequently, there has been a growing shift towards a fiber-rich diet, abundant in fruits, vegetables, seeds, and nuts, to enhance longevity and quality of life. The primary bioactive components in these plant-based foods are polyphenols, which exert significant effects on modulating the gastrointestinal microbiota through their antioxidant and anti-inflammatory activities. This modulation has preventive effects on neurodegenerative, metabolic, and cardiovascular diseases, and even cancer. The antimicrobial properties of polyphenols against pathogenic bacteria have significantly reduced the need for antibiotics, thereby lowering the risk of antibiotic resistance. This paper advances the field by offering novel insights into the beneficial effects of polyphenols, both directly through the metabolites produced during digestion and indirectly through changes in the host’s gastrointestinal microbiota, uniquely emphasizing swine as a model highly relevant to human health, a topic that, to our knowledge, has not been thoroughly explored in previous reviews. This review also addresses aspects related to both other animal models (mice, rabbits, and rats), and humans, providing guidelines for future research into the benefits of polyphenol consumption. By linking agricultural and biomedical perspectives, it proposes strategies for utilizing these bioactive compounds as therapeutic agents in both veterinary and human health sciences.

## 1. Introduction

Polyphenols are secondary plant metabolites that form a large family of phytochemicals, characterized by aromatic rings with one or more hydroxyl groups [1]. In plants, polyphenols play a crucial role in protection against UV radiation, pathogen attacks, and insect defense [2,3]. They are abundant in a variety of plant foods, including fruits, vegetables, nuts, chocolate, and tea [4,5]. Despite their widespread presence in the diet, polyphenols have limited bioavailability and largely reach the colon in an almost unaltered state. In the colon, they are catabolized by the gastrointestinal microbiota, producing metabolites that exhibit a broad spectrum of bioactive properties, including antioxidant, anti-inflammatory, and anticarcinogenic effects, which are attributed to the phenolic groups in their chemical structures [1,6].

Recent studies have shown an inverse correlation between polyphenol consumption and the prevalence of various diseases, including cancer and neurodegenerative, cardiovascular, and metabolic disorders [7,8,9,10,11]. Polyphenols have also been observed to possess significant antimicrobial properties. Flavonols and flavones can inhibit the growth of *Staphylococcus* spp. bacteria, simultaneously preventing biofilm formation and neutralizing the toxicity of enterotoxins. This effect occurs through the inactivation of acyl homoserine lactone, which is crucial for quorum sensing [4,12,13]. Additionally, tannins are capable of precipitating proteins, which enables them to inactivate extracellular bacterial enzymes and chelate ions essential for bacterial growth [14]. Due to their robust antimicrobial properties, polyphenols can modulate the gastrointestinal microbiota of the host by fostering the growth of beneficial bacteria and suppressing pathogenic species [15,16].

Consequently, polyphenols promote the proliferation of beneficial bacteria such as *Lactobacillus* spp. and *Bifidobacterium* spp., playing a crucial prebiotic role [17]. The number of potentially pathogenic bacteria, including species from the genera *Clostridium* spp. (C. *histolyticum/C. perfringens*), *Pseudomonas* spp., *Enterobacteria* spp. (*E. coli*, *Salmonella* spp. spp.), *Enterococcus* spp., *Staphylococcus* spp., *Streptococcus* spp., *K. pneumoniae*, and *P. aeruginosa*, decreases with the intake of polyphenols [18,19]. Similarly, the alterations induced by polyphenols in the gastrointestinal microbiota impact the host’s health [20,21]. For instance, they enhance lactose tolerance and reduce the incidence of diarrhea and constipation [17].

Most polyphenols are metabolized by the gastrointestinal microbiota, and the majority of their therapeutic effects stem from this process. Once consumed, polyphenols are converted by the gastrointestinal microbiota into compounds with lower molecular weights, facilitating their absorption by the host organism [22]. Notable, among the bacteria that can metabolize polyphenols are *Prevotellaceae ramulus*, *Bifidobacterium* spp., and *Flavonifractor plautii* [23]. Additionally, the metabolites resulting from the digestion of polyphenols by the gastrointestinal microbiota, once absorbed into the body, can trigger beneficial cellular signaling pathways for the host. For instance, butyrate, produced by *Faecalibacterium prausnitzii* and *Prevotellaceae rectale*, exhibits potent anti-inflammatory effects [18]. Similarly, anthocyanins and resveratrol possess antiviral [24,25] and anti-thrombotic properties [26].

The consumption of polyphenols could serve as an alternative to conventional medications that target the colon. This is because traditional drugs often lose a significant portion of their pharmaceutical activity due to the acidic environment and enzymatic activity in the stomach, as well as the extensive metabolic processes in the small intestine. These factors can degrade or alter the active compounds, reducing their efficacy before they reach their intended site of action. In contrast, polyphenols bypass these challenges in the upper gastrointestinal tract and are delivered directly to the colon. In the colon, they remain largely intact and are metabolized by the gastrointestinal microbiota, which enhances their effectiveness and allows them to exert their therapeutic effects more efficiently [27].

### 1.1. Polyphenols and Gut Microbiota

The gastrointestinal (GI) microbiota, consisting of trillions of microorganisms including bacteria, fungi, archaea, protozoa, and viruses, is recognized as a key factor in animal and human health [28,29]. The primary function of the gastrointestinal microbiota is to maintain metabolic homeostasis and support the optimal functioning of the immune system [30]. It supports the proper functioning of various systems, particularly the digestive and immune systems [31,32]. It contributes to host health by providing nutrients and active biocompounds, synthesizing vitamins, fermenting probiotics, and preventing pathogen colonization [33]. Furthermore, the GI microbiota enhances mineral absorption, synthesizes neurotransmitters, and regulates satiety [28].

Polyphenols are effective in modulating the microbiota because at least 90% of the consumed polyphenols reach the gastrointestinal tract, directly impacting its composition [1]. Additionally, the interaction of polyphenols with the GI microbiota occurs when these compounds are metabolized by the microbiota’s enzymes. The metabolites produced through this process then influence the microbial composition of the GI microbiota [4,10]. Moreover, when exposed to polyphenols, the microbiota can metabolize them to produce a variety of bioactive compounds that have potent therapeutic effects [34]. The microbiota modulates numerous metabolic pathways through ongoing interactions between the signaling molecules it produces and the host organism. These molecules initiate various signaling pathways [35].

The composition of beneficial bacteria within the microbiota includes genera such as *Cytophaga-Flavobacterium-Bacteroides* spp. (which includes *Prevotella* spp. and *Porphyromonas* spp.) and Firmicutes (comprising *Bacilli* spp., *Clostridia* spp., *Erysipelotrichia* spp., *Limnochordia* spp., *Negativicutes* spp., *Thermolithobacteria* spp., and *Tissierellias* pp.) [30,36]. These species are chiefly responsible for producing short-chain fatty acids (SCFAs), which help reduce inflammation and are involved in synthesizing vitamins like those in the B complex and vitamin K. Furthermore, their production of acetates helps prevent the colonization of the colon by pathogenic agents [30,37].

Given that numerous diseases are linked to dysbiosis, innovative strategies to combat these conditions have included altering the microbiota using various approaches. These include the application of prebiotics, probiotics, synbiotics, postbiotics, and even fecal material transplantation, all of which have shown promising outcomes. This underscores the significant role of the microbiota in maintaining the overall health of the host [27]. Furthermore, the composition of the microbiota varies based on the host’s characteristics, including their genome, gender, and age. Nonetheless, diet is the most critical factor in determining the GI microbiota’s composition [27].

### 1.2. Polyphenols in Regulating Swine Microbiota

Recent studies have therefore concentrated on dietary modifications to reshape the microbiota, aiming to treat diseases effectively [27]. In this context, preclinical research encompassing in vitro, ex vivo, and in vivo animal experiments has demonstrated the efficacy of polyphenols in modulating the GI microbiota for therapeutic purposes. The immediate effects noted include antioxidant and anti-inflammatory activities [10]. Pigs have been recognized as the most suitable animal models for human studies in preclinical research, particularly because they closely mimic the human gastrointestinal microbiota. Experiments that include polyphenols in their diet have yielded promising results [38,39]. Consequently, pigs have become a critical model for studying the GI microbiota due to their anatomical and physiological similarities to humans. Furthermore, by adjusting their diet, researchers can gather detailed insights into potential changes that might similarly affect humans [40]. The ability of polyphenols to substitute for certain medications is particularly intriguing due to their demonstrated anti-inflammatory, antioxidant, antimicrobial, and antiparasitic properties in pigs, effects that have similarly been observed in humans [41]. Moreover, with the pork industry shifting towards natural additives due to restrictions on antibiotics and zinc oxide [42,43], there is a rising interest in utilizing polyphenol-rich feeds as replacements for antibiotics, zinc oxide, and growth promoters. This approach capitalizes on the bioactive properties of polyphenols within the industry [44]. Insights gained from these studies may also offer guidelines for potential therapeutic benefits for humans.

### 1.3. The Purpose of This Study

This review marks a novel contribution using swine as a model for investigating the antimicrobial, antioxidant, and anti-inflammatory properties of polyphenols, which, to our knowledge, has not been comprehensively addressed in prior reviews. It presents a detailed examination of these bioactive compounds’ mechanisms of action, encompassing all major polyphenol classes in swine, mice, rabbits, rats, and humans, thereby offering valuable cross-species comparisons that enhance its relevance for biomedical applications.

By employing swine as a model—owing to their close resemblance to human gastrointestinal physiology—this review aims to advance the field by exploring the beneficial effects of polyphenols through the modulation of the gut microbiota, yielding insights that are more readily translatable to human health. This review integrates agricultural and biomedical perspectives, underscoring the dual role of polyphenols as therapeutic agents for humans and as natural additives for livestock feed. It further highlights the metabolic transformations that polyphenols undergo when exposed to gut microbial enzymatic activity, along with the resulting metabolites’ roles in activating diverse cellular signaling pathways that prevent and combat various diseases.

Collectively, these findings offer novel insights into the mechanisms underlying polyphenol interactions within both the swine and human microbiota, underscoring their significance in advancing animal and human healthcare.

## 2. Materials and Methods

### 2.1. Consulting the Specialized Literature

This report was conducted in accordance with the Preferred Reporting Items for Systematic Review and Meta-Analysis (PRISMA) protocols [45]. This report highlights the therapeutic effect of polyphenols through the influence of the gastrointestinal microbiota in pigs, mice, and humans. This report focused on experiments that analyzed the beneficial effects of polyphenol ingestion. The databases consulted for obtaining references were PubMed, Scopus, and Web of Science. The search was based on keywords present in the article title or abstract. For pigs, the keywords used were “polyphenol microbiota pig” and “polyphenol microbiota pig therapy”, while for mice, rabbits, rats, and humans, the keywords were “polyphenol microbiota” and “polyphenol microbiota therapy”.

### 2.2. Inclusion and Exclusion Criteria

**Figure 1:**
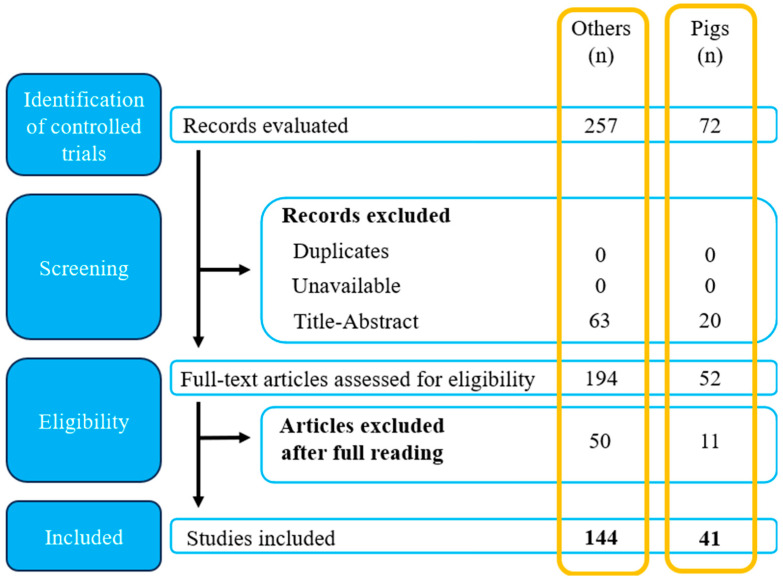
Diagram of the article selection process cited in this review.

The parameters for selecting articles were based on Population, Intervention, Comparison, Outcomes, and Study Design (PICOS), without restrictions regarding the year of publication. The selected studies included experiments conducted on pigs, mice, rabbits, rats, or humans, where polyphenols were administered orally under normal or pathological physiological conditions, with results focused on changes in the gastrointestinal microbiota and the therapeutic effects of polyphenol ingestion. The literature selection flow diagram (Figure 1) shows that 319 articles were analyzed, of which 257 were about humans and mice, rabbits, and rats, and 72 about pigs. No duplicate or unavailable articles were identified. Based on the content of titles, abstracts, and the PICOS table (Table 1), 63 articles about humans, mice, rabbits, and rats and 20 about pigs were excluded. Out of the remaining 246 articles, 50 about humans, mice, rabbits, and rats and 11 about pigs were eliminated after being read in full, based on the PICOS criteria. Thus, this study included 186 articles: 144 about humans, mice, rabbits, and rats, 41 about pigs, and 1 about the PRISMA protocol.

## 3. Results

### 3.1. Impact of Polyphenols on Gut Microbiota and Health in Pigs

Polyphenols are utilized in pig farming primarily as growth promoters, effectively reducing mortality rates [46]. This benefit is achieved by enhancing beneficial microbial species in the gastrointestinal microbiota and suppressing pathogenic ones. Pigs fed with polyphenols have exhibited an increase in the number of *Lactobacillus* spp. [46] and a reduction in the numbers of *Salmonella* spp. and *E. coli* [47]. Moreover, the antioxidant and anti-inflammatory properties of polyphenols facilitate optimal nutrient absorption. They also stimulate the secretion of digestive enzymes and foster the development of intestinal villi, which are crucial factors that significantly enhance pig growth [44].

Additionally, polyphenols have demonstrated the ability to alleviate the symptoms experienced by piglets during weaning [31]. This period involves a transition from a liquid to a solid diet, necessitating an adaptation phase that is often marked by a significant slowdown in growth rates. This slowdown is due to substantial changes in the morphology of the intestine and colon, as well as shifts in the composition of their microbiota [48,49].

Experiments involving polyphenols have revealed numerous health benefits for pigs, as detailed in Table 2. Incorporating polyphenols into pig feed, whether as isolated compounds from various plants or in blended mixtures, has demonstrated significant positive alterations to the microbiota, leading to therapeutic effects for pigs [31]. These benefits include improved gut health, enhanced immune response, and better overall growth rates. The introduction of polyphenols helps maintain a balanced gut environment, which is crucial during stress periods such as weaning, thereby reducing common issues like diarrhea and enhancing nutrient absorption and feed efficiency [44].

The benefits of polyphenols on pig health were researched by incorporating them into their feed. These polyphenols were added to the pigs’ diets in various forms, including tannin or phenolic acid enrichment, mixtures of multiple polyphenols, and the introduction of polyphenol-rich plants such as grapes, apples, or aronia.

#### 3.1.1. Types of Polyphenols

- Tannins

Supplementing pig feed with tannins extracted from gallnuts has been observed to increase the population of *Proteobacteria* spp. and decrease that of Firmicutes. It has also proven effective in reducing pathogenic species such as *Candidatus brocadia* and *Escherichia-Shigella*. In these pigs, antioxidant capacity was enhanced and intestinal inflammation was reduced, as indicated by lower levels of IL-1β and TNF-α in the jejunum [53]. Additionally, supplementation with isolated and hydrolyzed tannins from gallnuts increased the number of *Lachnospiraceae* spp. and *Prevotella* spp., which can degrade cellulose and hemicellulose, and *Lactobacillus amylovorus*, which can hydrolyze starch, while reducing the number of *Alloprevotella* spp., considered pathogenic. These changes led to decreased diarrhea frequency, enhanced antioxidant capacity with increased glutathione and superoxide dismutase levels, reduced serum malondialdehyde, and improved digestion and nutrient absorption due to their ability to digest complex polysaccharides [54]. Another approach to administering tannins is in a microencapsulated form. This method led to an increase in *Bacteroidetes* spp. and a decrease in Firmicutes. Furthermore, it improved duodenal morphology by enhancing the growth of intestinal villi, promoted protein absorption by upregulating the expression of genes *SLC15A1* and *SLC6A19* in the ileum, which encode proteins responsible for dipeptide and tripeptide transport, and improved colon function. Additionally, it helped protect the colon from pathogenic bacteria [55].

- Phenolic Acids

Phenolic acids or phenolcarboxylic acids are aromatic acid compounds. Supplementation of pigs’ feed with ellagic acid has been shown to increase the populations of *Ruminococcaceae* spp. and *Clostridium ramosum* while reducing numbers of the potentially pathogenic *Parabacteroides* spp. This polyphenol also enhances the intestinal barrier by boosting expression of junctional proteins like occludin, improving antioxidant activity in the jejunum, increasing SCFA concentrations, particularly acetic, propionic, and butyric acids, and stimulating carbohydrate metabolism and glycan synthesis [56]. Additionally, in pigs, ellagic acid has been noted to promote the growth of beneficial bacteria such as *Prevotella* spp. and *Lactobacillus delbrueckii* in the cecum and *Lactobacillus reuteri* in the rectum, which are known for their anti-inflammatory and antimicrobial properties, respectively. It also supports *Bifidobacterium* spp. and *Megasphaera* spp. in the cecum, which are involved in SCFA production. By acidifying the environment, ellagic acid aids in food fermentation and protection against pathogenic bacteria, reduces diarrhea frequency, increases concentrations of the junctional proteins zonula occludens (ZO-1) and occludin, boosts the immune system, and promotes fermentation by lowering pH [57].

Moreover, in pigs, supplementation with caffeic acid encourages the growth of *Alloprevotella* spp., *Prevotellaceae*, and *Prevotellaceae coprostanoligenes*, thus enhancing the overall health of the colon. This polyphenol promotes pig growth and enhances intestinal morphology and barrier function by upregulating the expression of claudin 1 and ZO-1. Additionally, it lowers serum levels of D-lactate and diamine oxidase, reduces pro-inflammatory cytokines (interleukin (IL)-1β, IL-6, tumor necrosis factor (TNF)-α), and alleviates oxidative stress. Furthermore, in pigs subjected to lipopolysaccharide (LPS) and caffeic acid treatment, there was an elevation in anti-inflammatory cytokines (IL-10), an increase in anti-apoptotic gene expression (Bcl-2), a decrease in pro-apoptotic gene expression (Bax, Fas), a decline in *Rikenellaceae* spp. abundance, and higher levels of bile acids and isovalerate, indicating an improvement in metabolic homeostasis [58].

Ferulic acid increases the *Firmicutes*/*Bacteroidetes* spp. ratio, beneficial for pig health, reduces the prevalence of *Prevotellaceae*, lowers cytokine levels associated with inflammation (IL-1β, IL-2, IL-6, TNF- α), decreases oxidative stress, and enhances the integrity of the intestinal barrier by boosting occludin levels in the ileum [43]. Supplementation with vanillic acid delivers benefits similar to those of ferulic acid but also increases populations of *Lachnospiraceae* spp., *Prevotellaceae eligens*, and *Prevotellaceae xylanophilum*, which help raise SCFA concentrations, particularly butyrate [43]. Chlorogenic acid has been found to increase populations of *Lactobacillus* spp., *Prevotella* spp., *Alloprevotella* spp., *Anaerovibrio* spp., and *Bifidobacterium* spp. It enhances the integrity of the intestinal barrier by reducing the activity of diamine oxidase and levels of major histocompatibility complex (MHC)-II in the jejunum and ileum, promotes fermentation by increasing concentrations of acetate, propionate, and butyrate in the cecum, and strengthens the intestinal epithelium by lowering D-lactic acid concentration and boosting claudin-1 expression in the jejunum and ileum. It also inhibits inflammation by reducing histamine levels and the number of tryptase-positive mast cells in the duodenum and jejunum and improves antioxidant capacity by increasing levels of catalase and glutathione peroxidase and reducing malondialdehyde [59,60]. Additionally, a decrease in pro-inflammatory cytokines (IL-1β, IL-6, TNF-α) and reductions in nuclear factor kappa-light-chain-enhancer of activated B cells (NF-kB), nuclear factor erythroid 2–related factor 2 (Nrf2), and heme oxygenase-1 (HO-1) indicate a significant reduction in inflammation and oxidative stress [59,60]. Protocatechuic acid increases the *Firmicutes*/*Bacteroidetes* spp. ratio and boosts populations of *Roseburia* spp., *Desulfovibrio* spp., *Megasphaera* spp., and *Fibrobacter* spp., associated with a healthy gut, while reducing numbers of *Prevotella* spp., *Holdemanella* spp., and *Ruminococcus* spp. torques, the latter two being linked with dysbiosis. It also helps reduce inflammation and oxidative stress by lowering IL-6, IL-2, and TNF-α levels and improves the function of the intestinal barrier by upregulating the expression of genes for claudin-1 and ZO-1 [61]. Thymol decreases the number of *Escherichia coli*, reduces the incidence of diarrhea, and enhances intestinal barrier function by decreasing jejunal permeability [62].

- Polyphenol Mixtures

Adding a blend of plant polyphenols with a high tannin concentration to pig feed resulted in an increase in *Lactobacilii* spp., contributing to a healthier intestinal environment [50]. Moreover, supplementing feed with GreenFIS polyphenols led to an increase in *Ruminococcus bromii*, which breaks down starch and produces short-chain fatty acids (SCFAs), thus enhancing the microbiota’s ability to utilize complex carbohydrates and improve nutrient absorption [51]. A Chinese herbal mix consisting of 42% Radix alba, 28% licorice, 28% dandelion, and 2% tea polyphenols has shown multiple therapeutic effects in pigs. This mix increased the population of Firmicutes, *Lactobacillus* spp., and *Blautia* spp., while reducing *Bacteroides* spp. and *Proteobacteria* spp. Additionally, it boosted antioxidant levels, enhanced immune responses by increasing IgM, white blood cells, neutrophils, and monocytes, decreased diarrhea frequency, and strengthened the integrity of the intestinal mucosa [79]. A different blend containing *Lonicera japonica*, *Astragalus membranaceus*, *Eucommia folium*, and *Codonopsis pilosula* had an opposite effect on GI microbiota composition, increasing and decreasing Firmicutes and *Clostridiales*. Despite these changes, the low ratio of Firmicutes to *Bacteroidetes* spp. preserved the therapeutic benefits, enhancing antioxidant activities and reducing ileum Keap1 protein levels, which is associated with heightened antioxidant effects [80]. Furthermore, a formulation comprising 0.75% quebracho and chestnut tannin extracts, 0.25% leonardite, and 0.2% tributyrin added to pigs’ basal diet has been shown to increase populations of *Lactobacillus* spp., *Bacteroidetes* spp., *Prevotellaceae* spp., and *Fibrobacteraceae* spp., while decreasing *Coliform* spp., Firmicutes, and *Chlamydiaceae* spp. This blend not only reduced diarrhea occurrence but also enhanced lipid metabolism, indicated by a decrease in low-density lipoproteins [52].

#### 3.1.2. Plants

- Grapes

Supplementation with grape seed meal in pig feed has been shown to increase the populations of *Prevotella* spp. and *Megasphaera* spp., significantly reducing inflammation and enhancing colon health through the promotion of fermentation and increased production of butyrate and isobutyrate, which fuel optimal colonic cell function [63]. Additionally, adding grape polyphenol extract and amino acids to the feed boosts *Lactobacilii aceae* numbers while reducing *Proteobacteria* spp. counts, also exhibiting anti-inflammatory effects by lowering NF-kB gene expression and lipopolysaccharide-binding protein levels. This supplementation aids in SCFA production, particularly increasing butyrate and propionate levels, and helps maintain metabolic homeostasis by lowering lactate, glucose, galactose, and hypoxanthine concentrations [64], and it may also decrease the incidence of diarrhea [65]. Grape extract further improves nutrient digestion [81,82]. Including grape pomace in the feed enhances the populations of *Lactobacillus delbrueckii*, *Olsenella umbonata*, and *Selenomonas bovis*, species known for their digestive benefits and protective roles against pathogenic bacteria. This supplementation also dampens the inflammatory response by reducing concentrations of pro-inflammatory cytokines (IL-1β, IL-8, IL-6, and TNF-α) and boosts immune system functionality through an increase in serum immunoglobulin G (IgG) levels [66]. However, in pigs infected with *Ascaris suum*, grape pomace supplementation has been associated with an increase in potentially pathogenic species like *Treponema* spp. and *Campylobacter* spp., and a decrease in beneficial *Lactobacillus* spp. and *Ruminococcus* spp., indicating that the effects of polyphenol supplementation can vary based on the health status of the pigs; in such cases, grape pomace supplementation may have detrimental effects. Nonetheless, the benefits are noted in the increase in eosinophils and SCFAs, both indicators of improved immune function and reduced inflammation [41]. Grape seed extract supplementation led to increased populations of *Lachnospiraceae* spp., *Clostridiales*, and *Ruminococcaceae* spp., which are effective in digesting complex carbohydrates and producing SCFAs like butyrate, and *Lactobacillus* spp., which helps prevent the colonization of the colon by pathogenic bacteria. This enhances intestinal functions and lowers inflammatory responses [38]. Furthermore, a mix of grape seed and grape marc meal extracts reduced populations of *Streptococcus* spp. and *Clostridium* spp., showing antimicrobial activity against pathogenic species. This mix also exhibits anti-inflammatory properties by downregulating several pro-inflammatory genes, such as intercellular adhesion molecule 1 (ICAM1), IL-1B, IL-8, and TNF, and supports the digestion process by lowering concentrations of volatile fatty acids like acetic, propionic, and valeric acids [67].

- Apples

Supplementation with apple pomace in pig feed has been shown to increase populations of *Bacteroidetes* spp., *Clostridia* spp., Firmicutes, and *Ruminococcaceae* spp. This addition also enhances intestinal morphology by promoting the growth of intestinal villi [68]. Furthermore, incorporating apple pectin into the feed leads to a rise in the numbers of *Anaeroplasma* spp., *Anaerostipes* spp., *Faecalibacterium* spp., *Prevotella* spp., and *Oscillibacter* spp., microbes beneficial to the GI microbiota that aid in fermentation processes. It also results in a reduction in potentially pathogenic species such as *Lawsonia* spp., *Staphylococcus* spp., *Aeromonas* spp., *Bacteroides* spp., and *Proteobacteria* spp. Additionally, apple pectin displays anti-inflammatory effects by lowering the expression of pro-inflammatory cytokines (IL-6, IL-8, IL-12, and IL-18) and boosts intestinal immunity by increasing the expression of mucin 2 (MUC2) and trefoil factor (TFF) genes and enhancing IgA secretion. The increased concentration of SCFAs further indicates enhanced digestive processes [13].

- Aronia

Supplementation with *Aronia melanocarpa* pomace resulted in a notable increase in beneficial bacteria such as *Lachnospira* spp., *Solobacterium* spp., *Romboutsia* spp., *Robinsoniella* spp., and *Prevotella* spp., which are linked to healthy gut function. Conversely, it led to a decrease in potentially pathogenic bacteria such as *Escherichia Shigella*, *Pseudoscardovia* spp., and *Proteobacteria* spp. This supplementation also enhanced intestinal barrier function by elevating concentrations of SCFAs like butyric, propionic, and caproic acid. It further improved the integrity of the intestinal barrier by increasing the gene expression of junction proteins such as claudin-1, occludin, and ZO-1, while simultaneously reducing inflammation through decreased expression of pro-inflammatory cytokines (IL-1β, IL-6, IL-8, and TNF-α) [69]. Additionally, *Aronia melanocarpa* pomace supplementation boosted the antioxidant status of the liver by enhancing the gene expression of glutathione peroxidase 1 (Gpx1) and (Gpx4), key enzymes in oxidative stress defense [70].

- Other plants

Supplementation with polyphenol concentrate derived from olive mill wastewater has demonstrated substantial antioxidant effects, significantly reducing serum concentrations of thiobarbituric acid reactive substances and protein carbonyls, alongside a decrease in diarrhea incidence [71]. Supplementation with rosemary extract boosted populations of *Bifidobacterium* spp. and *Bacteroidetes* spp., while reducing *Escherichia coli* counts. It has enhanced antioxidant defenses by elevating superoxide dismutase and glutathione peroxidase levels in the serum and liver, further contributing to a reduction in diarrhea occurrence [72]. Supplementation with purple sweet potato polyphenols led to increases in beneficial bacteria such as *Lactobacillus* spp., *Bifidobacterium* spp., *Prevotella* spp., and *Collinsella stercoris* in the gut microbiota, while reducing populations of *Clostridium* spp., *Proteobacteria* spp., and *Acidaminococcus* spp.—the latter of which produces ammonia from protein fermentation and can be toxic in high concentrations. Purple sweet potato polyphenols also support improved fermentation by lowering pH and decreasing the concentration of p-cresol, which reduces putrefactive by-products from protein fermentation [73]. Supplementation with cocoa husk meal raised levels of *Bacteroidetes* spp., *Prevotella* spp., and *Faecalibacterium prausnitzii*, and lowered levels of Firmicutes, *Lactobacillus* spp., *Enterococcus* spp., and *Clostridium histolyticum*. Despite a reduction in some beneficial bacterial species, this change led to a favorable intestinal microbial balance by increasing SCFAs concentrations [74]. Conversely, another study found that supplementation with flavanol-enriched cocoa powder increased populations of *Lactobacillus* spp. and *Bifidobacterium* spp., benefiting the immune system by reducing Toll-like receptor 9 (TLR9) gene expression, contributing to inflammation reduction, and lowering oxidative stress by increasing the O-methyl-epicatechin glucuronide metabolite [66]. Supplementation with Red osier dogwood polyphenol extract increased numbers of *Lactobacillus delbrueckii* and *Lactobacillus mucosae*. By elevating SCFA concentrations, particularly propionate, it supports optimal intestinal barrier function, enhances the immune system, and inhibits the growth of pathogenic bacteria [75]. Supplementation with mulberry leaves decreased numbers of *Olsenella* spp. and *Megasphaera* spp., reducing skatole concentrations, while also improving fermentation by increasing SCFA levels [76]. Supplementation with phytolina, a polyphenol from sugar cane, raised levels of beneficial *Lactobacillus* spp. and *Catenibacterium* spp. while reducing potentially pathogenic species such as *Monibacterium* spp., *Dialister* spp., and *Escherichia-Shigella*. It also enhanced fermentation, intestinal barrier integrity, and inflammation reduction, and led to increases in SCFA concentrations, such as propionic, vanillic, and propanoic acids, as well as elevated levels of luteolin, indicating the microbiota’s capacity to degrade complex polyphenolic compounds [77]. Supplementation with *Mountan cortex radicis*, rich in paeonol, increased levels of beneficial *Lactobacillus* spp. and Firmicutes, and decreased levels of potentially pathogenic *Bacteroides* spp., *Parabacteroides* spp., *Lachnospiraceae* spp., and *Enterococcus* spp. Additionally, it improved antioxidant status and reduced inflammation by lowering the expression of pro-inflammatory cytokine genes (interferon γ, TNF-α, IL-1β) and reducing NF-κB gene expression, while also increasing concentrations of SCFAs like acetic, butyric, and valeric acids [78].

### 3.2. Therapeutic Effects of Polyphenols on Mice, Rabbits, Rats, and Humans

**Figure 2:**
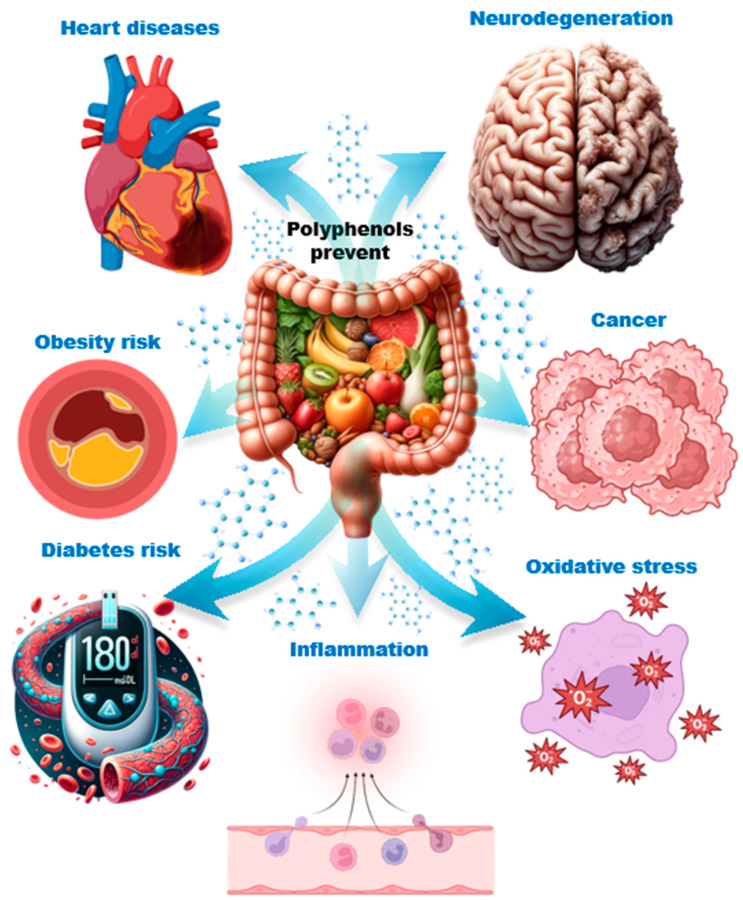
Beneficial effects of polyphenols in mice, rabbits, rats, and humans.

The metabolism of polyphenols by the gastrointestinal microbiota involves their breakdown into smaller bioactive compounds with reduced molecular weights, facilitating absorption and enabling their therapeutic effects [34]. The metabolism of ingested polyphenols can vary significantly depending on the specific microorganisms present in the GI microbiota. As a result, individuals consuming the same type of polyphenol may experience different therapeutic outcomes based on the ability of their intestinal microbiota to metabolize these compounds and produce therapeutic metabolites (Figure 2 and Table 3). This variation has led to the proposal of the term “metabotypes” [83]. Additionally, the way polyphenols are metabolized is influenced by the food matrix [84,85,86]. Genetic differences among individuals, particularly concerning molecular transporters and enzymes that process polyphenols and their metabolites, also play a critical role in their therapeutic efficacy [87,88]. The therapeutic effects of polyphenols on humans have been observed by monitoring changes in the gastrointestinal microbiota and measuring alterations in blood metabolites following the consumption of polyphenol-rich products. The most studied polyphenols include isoflavones (metabolized into daidzein), flavan-3-ols, flavones, flavanones, lignans, ellagitannins, ellagic acid, and resveratrol.

#### 3.2.1. Types of Polyphenols

- Isoflavones

**Figure 3:**
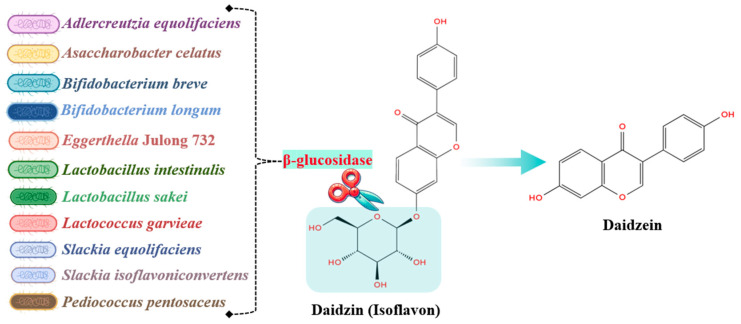
Hydrolysis of isoflavones into daidzein.

Isoflavones possess a 3-phenylchromen-4-one core structure with various substituents like glycoside, hydroxyl, or methoxyl groups [157]. After ingestion, isoflavones are hydrolyzed by the gastrointestinal (GI) microbiota into daidzein [90] (Figure 3). Depending on individual variations in the GI microbiota, daidzein may or may not be further metabolized into Equol and O-desmethylangolensin (ODMA). This leads to the identification of three distinct metabotypes: non-producers, who cannot metabolize daidzein; equol producers, who convert daidzein into equol; and equol + ODMA producers, who can metabolize daidzein into both compounds [89]. The microorganisms involved in daidzein metabolism include *Eggerthellaceae* spp., *Adlercreutzia equolifaciens*, *Adlercreutzia mucosicola*, *Slackia isoflavoniconvertens*, *Slackia equolifaciens*, *Enteroscipio* spp., *Lactococcus garvieae*, *Bifidobacteriaceae* spp., *Coriobacteriaceae* spp., *Asacchharobacter* spp., and *Clostridium* spp. [27]. Studies indicate that equol producers, characterized by high populations of *Adlercreutzia equolifaciens* and *Bifidobacterium bifidum*, exhibit a reduced diversity of GI microbiota, particularly in *Bacteroides* spp., *Faecalibacterium* spp., and *Butyrivibrium* spp. [158,159]. Equol producers are also at lower risk for aortic and coronary artery calcification [27] and arterial stiffness [93]. Moreover, synthetic equol administration has been shown to improve vascular function in equol producers, evidenced by reduced total cholesterol levels [92] and enhanced vascular function after isoflavone consumption [160]. Notably, the equol + ODMA metabotype is associated with the lowest risk of cardiometabolic diseases [94], and both equol and ODMA show anti-atherogenic effects [95]. Additionally, there is a noted association between obesity and the non-producer metabotype [91]. In rats, a diet lacking isoflavones significantly reduces the diversity of microbial species within the gut microbiota. This deficiency leads to an increased relative abundance of *Firmicutes*, including genera such as *Lactobacillus* and *Bifidobacterium*, while decreasing *Bacteroidetes* often associated with constipation and metabolic disorders. Another drawback of an isoflavone-deficient diet is the proliferation of pathogenic bacteria from the genus *Desulfovibrio*, which are linked to inflammation and irritable bowel syndrome. Additionally, the absence of isoflavones downregulates pathways involved in glycan biosynthesis, thereby hindering carbohydrate metabolism [96].

- Flavan-3-ols

Flavan-3-ols, also known as flavanols, are compounds derived from flavans, characterized by a 2-phenyl-3,4-dihydro-2H-chromen-3-ol core structure [161]. The microorganisms involved in the metabolism of flavan-3-ols include *Lactiplantibacillus plantarum* IFPL935, *Eggerthella lenta*, *Adlercreutzia equolifaciens*, and *Flavonifractor plautii* [27]. Research has shown that supplementation with cocoa flavan-3-ols results in a notable increase in the populations of *Lactobacillus* spp. and *Bifidobacterium* spp., along with a significant decrease in *Clostridium* spp. levels. Additionally, this supplementation has been linked to reductions in triglyceride and C-reactive protein levels [102]. Flavonols also promote the growth of beneficial bacteria such as *Bifidobacterium* spp., *Lactobacillus* spp., and *Akkermansia* spp., which may help prevent colon cancer by inhibiting pro-inflammatory cytokines, controlling angiogenesis, and cell proliferation [162]. The consumption of flavan-3-ols has been associated with enhanced vascular function, improved serum cholesterol profiles, and better glucose metabolism [27,97]. Moreover, flavan-3-ol intake has been linked to a decreased incidence of Alzheimer’s disease (AD) and neurodegeneration [99,100] and a reduction in the occurrence of prostate cancer [101]. Phenyl-γ-valerolactones, metabolites of flavan-3-ols, are known to inhibit amyloid plaque formation, prevent memory impairment, and reduce neuroinflammation [98]. In case of rats, anthocyanins derived from purple sweet potatoes increased the abundance of *Dorea*, a bacterial genus associated with enhanced carbohydrate digestion and positive effects on the host. Conversely, they reduced the populations of *Scillospira* and *Bacteroides*, which are linked to unfavorable metabolic profiles. Furthermore, grape seed proanthocyanidin extract elevated levels of hippuric acid and 3′-4′-dihydroxycinnamic acid, promoting improved antioxidant and anti-inflammatory responses [103,104].

- Flavones and Flavanones

Flavones are characterized by a backbone structure of 2-phenylchromen-4-one (2-phenyl-1-benzopyran-4-one) [163]. The primary structural distinction between flavones and flavanones lies in the saturation of the C ring, which is saturated in flavanones [164]. Among flavones, hesperidin (hesperetin-7-O-rutinoside) is metabolized in the colon by microbes such as *Bifidobacterium* spp., *Clostridium* spp., *Bacteroides* spp., *Lactobacillus* spp., and *Prevotellaceae* spp. [110]. A strategic therapeutic use of polyphenols involves their incorporation into synbiotics, which combine prebiotics and probiotics. This combination allows the selected probiotic strain to utilize a prebiotic substrate for synthesizing beneficial metabolites aimed at achieving specific therapeutic effects [109,112]. Common prebiotics used in synbiotics include carbohydrates and polyphenols, with *Lactobacillus* spp. and *Bifidobacterium* spp. being the probiotics most frequently employed [27]. An example is a synbiotic containing the probiotic *Bifidobacterium longum* and flavanones as prebiotics, which enhances the bioavailability of flavanones, increasing blood concentrations of metabolites like esperetin-O-glucuronides, naringenin-O-glucuronides, and hesperetin-30-O-sulfate, thus enhancing their therapeutic effects [111]. Flavones and flavonols are linked with a reduced risk of coronary heart disease (CHD) [97], and flavonoid consumption has been correlated with a 50% reduction in dementia risk [106]. Additionally, metabolites from cocoa enriched with flavonoids have been shown to enhance the function of the hippocampal dentate gyrus through improved vascularization [108]. Luteolin encourages the growth of SCFA-producing bacteria such as *Bifidobacterium* spp. and *Bacteroides* spp. and is suggested to reduce cancer cell proliferation [107]. Quercetin promotes the growth of *Akkermansia muciniphila* while inhibiting *Enterococcus* spp., potentially preventing the onset of amyotrophic lateral sclerosis (ALS) [30]. Myricetin stabilizes E6-AP, which is involved in neurodevelopment, offering protection against neurodegenerative diseases by modulating the microbiota, specifically by increasing *Actinobacteria* spp. and *Verrucomicrobia* spp. and decreasing *Proteobacteria* spp. and *Bacteroidetes* spp. [113,114]. In rats, apigenin-7-glucoside, a flavone, is metabolized by *Eubacterium ramulus* into apigenin, which is subsequently broken down by *Clostridium orbiscindens* into phenolic acids. Additionally, *Bacteroides distasonis* and certain *Escherichia coli* species also participate in its metabolic process. The health benefits of apigenin-7-glucoside consumption stem from its antioxidant, anti-inflammatory, and antimutagenic properties [105].

- Lignans

**Figure 4:**
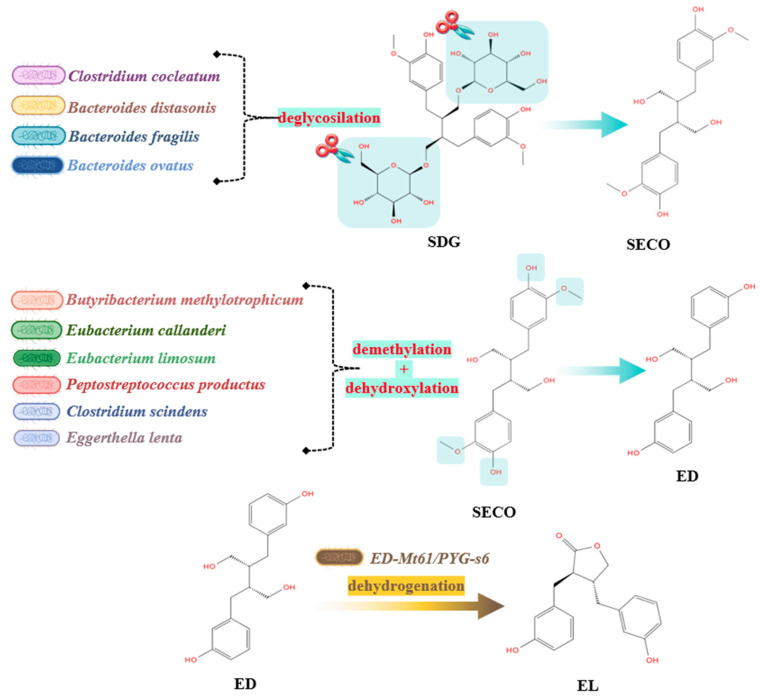
Metabolization of lignans into enterolignans.

Lignans typically exhibit dimeric structures, characterized by a β,β’-linkage connecting two phenylpropane units [165]. Lignans are metabolized into enterolignans, specifically enterolactone (EL) and enterodiol (ED), through a multi-step process [118] (Figure 4). Initially, lignans are converted to secoisolariciresinol diglucoside (SDG), which is then transformed into secoisolariciresinol (SECO) by the action of various bacteria including *Bacteroides distasonis*, *Bacteroides fragilis*, and *Bacteroides ovatus*, as well as *Clostridium cocleatum* and *Clostridium* spp. [115]. SECO is further demethylated by microbes such as *Butyribacterium methylotrophicum* and *Prevotella limosum* and undergoes dihydroxylation by *Clostridium scindens* and *Eggerthella lenta* to produce ED. EL is then generated through the dehydrogenation of ED [166]. Not everyone can produce EL, creating a distinction between metabotypes: ED producers and EL producers [115]. EL producers are known to exhibit increased microbial diversity, specifically with a higher presence of *Faecalibacterium prausnitzii* and *Alistipes shahii* [119]. Furthermore, EL production has also been linked to increases in populations of *Moryella* spp. and *Acetanaerobacterium* spp. [116]. Additionally, certain strains of *Lactobacillus* spp. and *Bifidobacterium* spp. can convert SECO into ED and EL [121]. Cohort studies have shown that both EL and ED are inversely associated with obesity or weight gain, and they influence estrogen signaling, lipid, and bile acid metabolism [118,123]. ED, specifically, has been linked to decreased weight gain [167]. Moreover, EL levels have been inversely related to obesity and other markers of cardiometabolic diseases such as elevated triglycerides, glucose, and insulin levels [123]. Due to their structural similarity to 17β-estradiol, lignans can bind to estrogen receptors and exhibit either estrogenic or antiestrogenic activities [117]. They are also noted for their protective effects against cardiovascular diseases [122]. A high lignan-to-EL conversion phenotype correlates with an anti-inflammatory status, characterized by the inhibition of NF-κB and nitric oxide synthase 2 (NOS2) and the upregulation of peroxisome proliferator-activated receptor gamma (PPARγ) [120].

- Ellagitannins and Ellagic Acid

Ellagitannins are a type of hydrolyzable tannin composed of a linear chain of gallic acid molecules esterified to glucose [168]. Ellagitannins and ellagic acid, once ingested, may or may not be metabolized into urolithins depending on the individual’s gut microbiota, resulting in three metabotypes: UM0 (non-producers), UMA (producers of urolithin A), and UMB (producers of urolithin B and isourolithin A) [27]. Specific gut microbial species such as *Gordonibacter pamelaeae* and *G. urolithinfaciens*, along with *Ellagibacter isourolithinifaciens* from the *Eggerthellaceae* spp. family, are responsible for converting ellagic acid into urolithins [27]. The genus *Gordonibacter* spp. is associated with higher levels of urolithin A, whereas it shows an inverse correlation with urolithin B and isorolithin A production [138]. Additionally, *Enterocloster bolteae*, *Bifidobacterium pseudocatenulatum*, *Lactococcus garvieae*, *Enterococcus faecium*, and *Streptococcus thermophilus* have also been linked to urolithin A production [10]. Differences in gut microbial ecology between urolithin metabotypes are evident. The genera *Gordonibacter* spp., *Paraeggerthella* spp., and *Eggerthella* spp. correlate with the UMA phenotype, while *Ellagibacter* spp., *Olsenella* spp., *Senegalimassilia* spp., *Slackia* spp., and *Adlercreutzia* spp. correlate with UMB [129]. *Olsenella* spp., *Senegalimassilia* spp. and *Slackia* spp. are also corelated with isourolithin A and urolithin B production [134]. UMA enhances the growth of *Akkermansia muciniphila*, inversely correlated with obesity, and improves dysregulation of phosphatidylinositol 3-kinase–protein kinase B (PI3K-Akt) in prostate cancer patients [26,133,139] UM0 is characterized by a low abundance of butyrate-producing microbial species and a high number of *Enterobacteriaceae* [10]. Urolithins are known to persist in the body for up to 80 h after consumption due to enterohepatic recirculation and the microbiota’s slow metabolism of ellagitannins and ellagic acid [20,130]. The prevalence of UMA and UMB metabotypes changes with age, with UMA decreasing in favor of UMB as age increases, while the frequency of UM0 remains consistent throughout life [126]. UMA is generally more favorable for weight management than UMB and UM0 [127]. UMB is linked to a higher risk of cardiovascular diseases and elevated low-density lipoprotein (LDL) cholesterol levels, whereas UMA is associated with increased high-density lipoprotein (HDL) cholesterol production [132,138]. Women with the UMA phenotype experience more significant postpartum weight loss and microbiome restoration compared to those with UMB [127]. Furthermore, UMB and UM0 are identified as risk factors for obesity in children, with UM0 also showing a higher prevalence of obesity [27,125]. Among obese individuals consuming ellagitannins, only those in the UMB group showed improvements in blood lipid profiles, potentially due to a poorer initial profile compared to the UMA group [20,130]. Both UMA and UMB exhibit beneficial cardiovascular and anti-inflammatory effects [128]. Moreover, obese individuals with the UMB metabotype who consumed pomegranate extract enriched with ellagitannins demonstrated improvements in cardiometabolic markers [20,130,131]. Additionally, UMB is frequently found in individuals with colorectal cancer and various metabolic diseases, which may indicate a dysbiosis origin [136,169]. Moreover, ellagic acid may possess anti-tumor activity in colon and breast cancer [170]. Also, in Parkinson’s disease, UM0 is the most prevalent metabotype and its frequency increases with the severity of symptoms [135]. In the case of rabbits, including chestnut and quebracho tannins in rabbit diets has been associated with a reduction in the phylum *Firmicutes*, particularly the families *Ruminococcaceae* and *Lachnospiraceae*. Tannins also decreased levels of proteolytic bacteria like *Clostridium perfringens* and pathogenic bacteria such as *Escherichia* coli. Among archaea, species from the family *Methanobrevibacter* were reduced. Notably, the populations of bacteria from the families *Bacteroides* and *Bifidobacterium* remained unaffected by tannin supplementation. This decrease in pathogenic species consequently lowers the risk of gastrointestinal diseases specific to rabbits, such as epizootic rabbit enteropathy [124].

- Resveratrol

**Figure 5:**
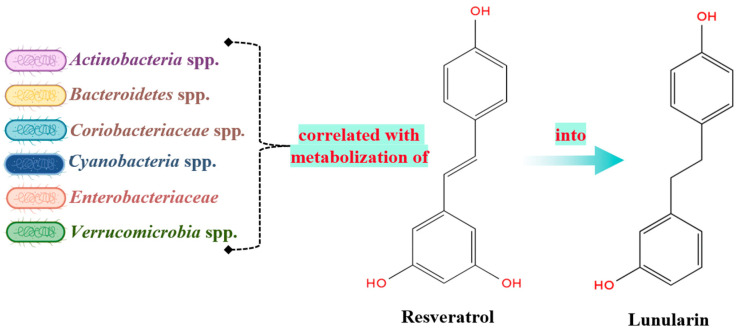
Metabolization of resveratrol into lunularin.

The structure of resveratrol comprises two phenolic rings connected by a double styrene bond, forming 3,5,4′-trihydroxystilbene [171]. Resveratrol is metabolized into dihydroresveratrol (DHR), 3,4′-dihydroxy-trans-stilbene (DHST), and 3,4′-dihydroxybibenzyl lunularin, (LUN) [144] (Figure 5). Individuals capable of metabolizing resveratrol into LUN show higher abundances of *Bacteroidetes* spp., *Actinobacteria* spp., *Verrucomicrobia* spp., *Cyanobacteria* spp., *Enterobacteriaceae*, and *Coriobacteriaceae* spp., whereas DHR producers tend to have high numbers of *Slackia equolifaciens* and *Adlercreutzia equolifaciens* [144]. LUN producers may be associated with a reduced risk of certain diseases, indicating that the metabolism of resveratrol to produce LUN reflects a healthy and functional microbiota that contributes to immunity and nutrient absorption. Additionally, LUN producers have been linked to enhanced anticarcinogenic and anti-inflammatory activities [88]. *Bifidobacterium infantis* is another species capable of metabolizing resveratrol. The metabolites produced exhibit potent anti-inflammatory and antioxidant effects by modulating the expression of the Sirtuin-1 pathway (SIRT-1), NF-κB, and Nrf2 [22]. Resveratrol consumption also increases populations of *Bacteroides* spp., *Akkermansia muciniphila*, *Butyrivibrio fibrisolvens*, and other resveratrol-responsive microbiota such as *Lachnospiraceae* NK4A136, *Blautia* spp., *Lachnoclostridium* spp., *Parabacteroides* spp., and *Ruminiclostridium* spp., which are typically reduced in ALS patients [30]. Furthermore, resveratrol promotes microbiota that favor the production of SCFAs, leading to an anti-inflammatory T cell response. This includes histone deacetylases (HDAC) inhibition, increased expression of the transcription factor forkhead box P3 (FoxP3) specific to regulatory T cells (Tregs), and the anti-inflammatory cytokine IL-10, which help mitigate inflammation associated with colorectal cancer [143]. Moreover, resveratrol also regulates p53 expression in breast cancer [146,147]. Through these mechanisms, resveratrol modulates cellular signaling pathways related to cell proliferation, apoptosis, autophagy, inflammation, and immune response, significantly exerting significant anti-tumor effects [148]. For rats, resveratrol increased the numbers of *Allobaculum, Ruminococcus, Clostridium sensu stricto, Bacteroides, Eubacterium*, and *Ligilactobacillus murinus*, while reducing *Psychrobacter*. It also reduced bacteria from the genus *Bilophila*, associated with adverse bile acid metabolism. Resveratrol lowered pro-inflammatory markers like TNF-α and IL-1β and increased anti-inflammatory cytokines such as IL-10. It upregulated tight junction proteins like ZO-1 and occludin, boosted antioxidant markers like superoxide dismutase (SOD), and decreased pro-oxidant enzymes like myeloperoxidase (MPO). By regulating inflammatory pathways, it may prevent or alleviate conditions like colitis or non-steroidal anti-inflammatory drug-induced gut injuries. Additionally, resveratrol propionate esters (RPE2 and RPE4) significantly reduced hypertension in a juvenile rat model of adenine-induced chronic kidney disease and ameliorated CKD-induced alterations in gut microbiota composition [140,141,142].

#### 3.2.2. Beneficial Properties of Polyphenols

- General Positive Effects of Polyphenols

The therapeutic effects of polyphenols are linked to their ability to activate various cellular signaling pathways, enhancing the host’s antioxidant and anti-inflammatory responses and mitigating the development of cardiovascular diseases [5]. The metabolites produced play critical roles in regulating numerous cellular processes, including immune system modulation, oxidative stress response, inflammation, glucose and lipid homeostasis, and DNA repair [172]. One specific mechanism involves the activation of the PI3K-Akt and endothelial nitric oxide synthase (eNOS) kinases, which increase nitric oxide levels. This nitric oxide then stimulates guanylate cyclase in blood vessel muscles, increasing cyclic guanosine 3′,5′-monophosphate concentration, which activates protein kinase G, leading to vasodilation and improved blood flow [173,174,175]. Additionally, PI3K-Akt pathway activation promotes Nrf2 expression, which is crucial in managing oxidative stress [176] and is inversely related to the risk of atherosclerosis [60]. Polyphenol consumption is also associated with increased levels of *Bifidobacterium* spp., which correlates with improved cholesterol levels [5]. Supplementation with aronia has been shown to increase populations of *Anaerostipes* spp., *Lawsonibacter asaccharolyticus*, and *Intestinimonas butyriciproducens*, all of which are linked to SCFA production [155,156]. SCFAs, facilitated by the presence of *Bifidobacterium* spp. [177], can inhibit histone deacetylation in microglial cells, reducing pro-inflammatory cytokines such as IL-1ß, IL-6, and TNF-α while increasing anti-inflammatory cytokines like transforming growth factor TGF-β and IL-4 [178]. This histone deacetylation enhances cognitive functions, memory, and may prevent neurodegenerative diseases by decreasing amyloid-β protein expression and tau protein phosphorylation, both implicated in neurodegeneration [177]. SCFAs also show anti-tumor activity in colorectal cancer and lymphoma by blocking histone deacetylation, significantly exerting anticarcinogenic effects [179]. Further studies reveal that polyphenol consumption boosts the number of *Faecalibacterium prausnitzii* and *Akkermansia* spp., known for their anti-inflammatory butyrate production [180]. Moreover, increased levels of metabolites such as 3,4-dihydroxyphenylacetic (3,4 DHPA) and 3-hydroxyphenyl acetic (3 HPA) acids, produced by the GI microbiota following polyphenol intake, activate cellular signaling pathways in glial cells, reducing neuroinflammation [180]. In lung cancer patients, a reduction in butyrate-producing bacteria such as *Clostridium leptum* and *Faecalibacterium prausnitzii* has been observed, alongside altered microbiota compositions with increases in *Kluyvera* spp. and *E. coli*, and decreases in *Veillonella* spp., *Fusobacterium* spp., and *Bacteroides* spp. compared to healthy individuals [181,182]. Furthermore, the consumption of polyphenol-rich fruits influences the gastrointestinal microbiota, impacting cancer prevention. A notable correlation has been found between non-consumption of fruits and decreased levels of *Prevotella* spp., another SCFA-producing bacterium, in bladder cancer patients [1]. For rats, polyphenols from *Aronia melanocarpa* increased *Lactobacillus* and *Ruminococcaceae* and decreased *Proteobacteria*. They upregulated tight junction proteins like ZO-1, occludin, and claudin-1, reducing intestinal permeability and preventing bacterial translocation. Additionally, they lowered cytokines such as IL-6, IL-1β, and TNF-α and reduced gut-derived endotoxins like lipopolysaccharide (LPS), protecting the liver from inflammation and oxidative damage [154].

- Polyphenols and Cancer

Polyphenols exhibit prebiotic effects by promoting the growth of beneficial bacteria and suppressing pathogenic ones in the gastrointestinal microbiota [20,130]. Pathogens such as *Escherichia coli*, *Clostridium perfringens*, and *Helicobacter pylori* are inhibited by polyphenols, which helps in the management of various gastrointestinal issues like diarrhea and irritable bowel syndrome [21,183]. *Helicobacter pylori*, known for producing oncogenic metabolites CagA and cytotoxin A that trigger gastric cancer, is effectively targeted by polyphenols, providing an anticancer benefit [1]. Furthermore, polyphenols enhance the effectiveness of the immune response by increasing the population of *Lactobacilii* spp., whose metabolites activate immune cells like natural killer cells and dendritic cells, aiding in the elimination of potential cancer cells [184,185]. The antimicrobial action against *E. coli* also lowers cancer risk due to its production of colibactin, a genotoxin associated with DNA damage [186]. Moreover, polyphenols from palm dates have been shown to reduce the risk of colon cancer [187]. Additionally, epigallocatechin gallate (EGCG) is recognized for its effectiveness in cancer prevention [188], and curcumin is noted for its anticancer properties, particularly against lung cancer [189]. Curcumin also effectively reduces colon tumor size [190]. Furthermore, catechins from green tea increase *Bifidobacterium* spp. populations, which, as producers of SCFAs, play a role in reducing inflammation and thereby slowing carcinogenesis by inhibiting NF-κB, TNF-α, and IL-6 expression [152]. In the case of rats, the combination of quercetin and catechin increased the number of Bacteroidetes and reduced harmful groups like *Proteobacteria*. Catechin specifically decreased the numbers of *Firmicutes, Ruminococcaceae, Lachnospiraceae*, and *Bifidobacteria*, while increasing *Prevotella, Parabacteroides*, and *Fusicatenibacter*. It significantly elevated propionate levels, associated with improved energy metabolism and appetite regulation, and raised serum leptin levels, correlating with a reduction in body weight [153].

- Polyphenols and Neurological Diseases

The gastrointestinal microbiota significantly influences the central nervous system (CNS) through the gut–brain axis, mediated by hormones, cytokines, and neurotransmitters. This interaction is crucial for the development and activation of microglia via metabolites produced by the microbiota and absorbed by the host [191,192]. Communication between the GI microbiota and the CNS occurs through various pathways including neuronal, vascular, immune, and neuroendocrine systems. These metabolites are pivotal in addressing numerous neurodegenerative diseases such as Alzheimer’s disease (AD), Parkinson’s disease (PD), Huntington’s disease (HD), multiple sclerosis, and mental disorders including schizophrenia, autism, depression, and anxiety. Dysbiosis in the GI microbiota has also been linked to the onset of these neurodegenerative diseases [192]. In PD patients, there is a notable decrease in *Faecalibacterium* spp. and *Anaerostipes* spp. and an increase in pro-inflammatory *Escherichia coli* and *Klebsiella* spp. [135]. EGCG supplementation reduces CsgA levels, a protein that contributes to α-synuclein amyloid aggregation implicated in PD, thus aiding in PD prevention [30,193]. Additionally, supplementation with curcumin, quercetin, and proanthocyanidins promotes an increase in *Bacteroides* spp., *Bifidobacterium* spp., and *Lactobacillus* spp., while reducing populations of *Escherichia* spp. and *Enterobacter* spp. This microbial balance supports gamma-aminobutyric acid (GABA) production, which is crucial as patients with ALS show a reduction in beneficial bacteria [30,194]. Pterostilbene and caffeic acid enhance levels of *Akkermansia* spp. and *Odoribacter* spp., which inhibit monoamine oxidase B (MAOB), thereby increasing serotonin levels, which are typically low in ALS [30,151]. In rats, pterostilbene supplementation decreases the abundance of *Firmicutes* while increasing the *Verrucomicrobia* phylum, particularly species such as *Akkermansia muciniphila* and *Odoribacter splanchnicus*. These microbial shifts are associated with weight loss in obese rats. Furthermore, pterostilbene stimulates the production of butyric acid, which supports a healthy lipid profile and overall metabolic well-being [149].

## 4. Conclusions

Polyphenols are known to exert numerous therapeutic effects by modulating the host’s microbiota. Upon entering the gastrointestinal tract, these compounds interact with the microbiota, undergoing metabolism that produces beneficial metabolites. These metabolites, along with changes in the composition of the GI microbiota, contribute to a prophylactic effect against various conditions, including cancer, metabolic disorders, cardiovascular diseases, neurological disorders, chronic inflammation, and immune deficiencies. However, the effectiveness of polyphenols can be limited by individual differences in metabolism, which are influenced by each person’s unique metabotype. This results in variability in the bioavailability of polyphenols, thus affecting their therapeutic potential. Furthermore, the types of microbial metabolism of polyphenols depend on the species present within the GI microbiota, and these processes can significantly differ based on the individual’s sex and age. Such variability in bioavailability is a key limitation in using polyphenols as therapeutic agents. Nevertheless, the presence of polyphenol metabolites in the blood serves as a useful indicator of the diversity, health, functionality, and composition of the GI microbiota. These metabolites can act as markers for the health of the microbiota, and fluctuations in these markers can be monitored and correlated with changes in health status or shifts in the microbiota influenced by diet, age, disease, and other factors.

## Figures and Tables

**Table 1:** PICOS table of inclusion and exclusion criteria for articles cited in this review.

Parameters	Inclusion Criteria	Exclusion Criteria
**Population**	Humans, mice, rabbits, rats, and pigs	Other species
**Intervention**	Ingested polyphenols	Non-oral treatments
**Comparisons**	Physiological and pathological conditions	Studies without statistical analysis
**Outcomes**	Modulation of the gastrointestinal microbiota	Non-gastrointestinal microbiota studies
**Study design**	In vivo studies	In silico and in vitro studies

**Table 2:** Changes induced by polyphenols in the microbiota and their beneficial effects in pigs.

No. Crt.	Origin/Type of Polyphenols	Related GI Microbiota	Therapeutic Effect	Ref.
1	Mix of polyphenols, predominantly tannins	increase in *Lactobacillus* spp.	healthier gut microbiome	[50]
2	Mix of polyphenols, GreenFis	increase in *Ruminococcus bromii*	starch degradation	[51]
3	Polyphenolic extract	increase in *Lactobacillus* spp.	reduced incidence of diarrhea	[52]
	mixture	increase in *Bacteroidetes* spp.	positive modulation of lipid metabolism	
		increase in *Prevotellaceae* spp.		
		increase in *Fibrobacteraceae* spp.		
		decrease in *Coliform* spp.		
		decrease in *Firmicutes*		
		decrease in *Chlamydiaceae* spp.		
4	Gallnut tannic acid	decrease in *Proteobacteria* spp.	increase in antioxidant capacity	[53]
		decrease in *Firmicutes*	reduction in intestinal inflammation	
		decrease in *Candidatus brocadia*	enhancing the gut barrier function	
		decrease in *Escherichia-Shigella*		
5	Hydrolyzed Chinese	increase in *Lachnospiraceae* spp.	decrease in the diarrhea rate	[54]
	gallnut tannic acid	increase in *Prevotella* spp.	improvements in antioxidant capacity	
		increase in *Lactobacillus amylovorus*	improve nutrient digestion and absorption	
		decrease in *Alloprevotella* spp.		
6	Encapsulated tannic acid	increase in *Bacteroidetes* spp.	improvement in the duodenal morphology	[55]
		decrease in *Firmicutes*	promote absorption of di- and tripeptides and neutral amino acids	
			protect piglets against pathogenic bacteria	
			enhance gut barrier function	
7	Ellagic acid	increase in *Ruminococcaceae* spp.	enhancing intestinal barrier function	[56]
		increase in *Clostridium ramosum*	enhanced the antioxidant capacity	
		decrease in *Parabacteroides* spp.	increased metabolites like short-chain fatty acids	
		increase in *Lactobacillus delbrueckii*	with improved gut health and function	
		increase in *Lactobacillus reuteri*	enhanced carbohydrate metabolism capabilities	
		increase in *Bifidobacterium* spp.	lower diarrhea rate	[57]
		increase in *Megasphaera* spp.	improved intestinal barrier function	
		increase in *Prevotella* spp.	stimulated immune response	
			lower the intestinal pH	
8	Caffeic acid	increase in *Alloprevotella* spp.	improved the growth performance	
		increase in *Prevotellaceae* spp.	improved intestinal morphology and barrier function	[58]
		increase in *Prevotellaceae*	decreasing inflammatory cytokines	
		* coprostanoligenes *	increasing anti-inflammatory cytokines	
		decrease in *Rikenellaceae* spp.	decreased oxidative stress	
			decreasing the expression of pro-apoptotic genes	
			increasing the expression of anti-apoptotic gene	
			improvement in metabolic homeostasis	
9	Feluric acid	increase in *Firmicutes/Bacteroidetes* ratio	decreased serum levels of inflammatory cytokines	[43]
		decrease in *Prevotellaceae* spp.	reduced oxidative stress	
			improved gut barrier integrity	
10	Vanilic acid	increase in *Firmicutes/Bacteroidetes* ratio	decreased serum levels of inflammatory cytokines	[43]
		increase in *Lachnospiraceae*	reduced oxidative stress	
		increase in *Prevotellaceae eligens*	improved gut barrier integrity	
		increase in *Prevotellaceae xylanophilum*		
		decrease in *Prevotellaceae* spp.		
11	Chlorogenic acid	increase in *Lactobacillus* spp.	improved intestinal barrier integrity	
		increase in *Prevotella* spp.	enhanced fermentation	[59]
		increase in *Alloprevotella* spp.		
		increase in *Anaerovibrio* spp.		
		increase in *Bifidobacterium* spp.		
			suppressed inflammation	[60]
			enhanced antioxidant capacity	
12	Protocatechuic acid	increase in *Firmicutes/Bacteroidetes* ratio	reduction in oxidative stress and inflammationenhancement in intestinal barrier function	[61]
		increase in *Roseburia* spp.		
		increase in *Desulfovibrio* spp.		
		increase in *Megasphaera* spp.		
		increase in *Fibrobacter* spp.		
		decrease in *Prevotella* spp.		
		decrease in *Holdemanella* spp.		
		decrease in *Ruminococcus torques*		
13	Thymol	decrease in *E. coli*	reduced diarrhea incidence	[62]
			improved intestinal barrier function	
14	Grape seed meal	increase in *Prevotella* spp.	maintain gut barrier integrity	[63]
		increase in *Megasphaera* spp.	suppressed inflammation	
15	Grape extract	increase in *Lactobacillaceae* spp.	anti-inflammatory effects	[64]
	polyphenols and amino	decrease in *Proteobacteria* spp.		[65]
	acids		reduced diarrhea incidence	
16	Grape pomace	increase in *Lactobacillus delbrueckii*	a reduced inflammatory response	[66]
		increase in *Olsenella umbonata*	improvement in immune function	
		increase in *Selenomonas bovis*		
17	Grape seed extract	increase in *Lachnospiraceae* spp.	enhanced gut barrier function	[38]
		increase in *Clostridiales* spp.	reduced inflammatory response	
		increase in *Lactobacillus* spp.		
		increase in *Ruminococcaceae* spp.		
18	Grape seed and grape	decrease in *Streptococcus* spp.	potent anti-inflammatory properties	[67]
	marc meal extract	decrease in *Clostridium* spp.	decrease in volatile fatty acids	
19	Apple pomace	increase in *Bacteroidetes* spp.	increased villus length	[68]
		increase in *Clostridia* spp.		
		increase in *Firmicutes*		
		increase in *Ruminococcaceae* spp.		
20	*Aronia melanocarpa*	increase in *Lachnospira* spp.	improved intestinal barrier function	[69]
	pomace	increase in *Solobacterium* spp.	reduced inflammation	
		increase in *Romboutsia* spp.		
		increase in *Robinsoniella* spp.		
		increase in *Prevotella* spp.		
		decrease in *Escherichia Shigella*		
		decrease in *Pseudoscardovia* spp.		
		decrease in *Proteobacteria* spp.		
			improved antioxidant status	[70]
21	Olive mill wastewater		beneficial antioxidative effect	[71]
			reduced diarrhea incidence	
22	Rosemary extract	increase in *Bifidobacterium* spp.	reduced diarrhea incidence	[72]
		increase in *Bacteroidetes* spp.	enhanced antioxidant capacity	
		decrease in *Escherichia coli*		
23	Purple sweet potato	increase in *Lactobacillus* spp.	enhance fermentation	[73]
	polyphenols	increase in *Bifidobacterium* spp.		
		increase in *Prevotella* spp.		
		increase in *Collinsella stercoris*		
		decrease in *Clostridium* spp.		
		decrease in *Proteobacteria* spp.		
		decrease in *Acidaminococcus* spp.		
24	Cocoa husk meal	increase in *Bacteroidetes* spp.	favorable intestinal microbial balance	[74]
		increase in *Prevotella* spp.		
		increase in *Faecalibacterium prausnitzii*		
		decrease in *Firmicutes*		
		decrease in *Lactobacillus* spp.		
		decrease in *Enterococcus* spp.		
		decrease in *Clostridium histolyticum*		
25	Flavanol-enriched cocoa	increase in *Lactobacillus* spp.	supporting the immune system	[66]
		increase in *Bifidobacterium* spp.	reducing oxidative stress in the body	
26	Red oiser dogwood	increase in *Lactobacillus delbrueckii*	strengthening the gut barrier	[75]
	polyphenol extract	increase in *Lactobacillus mucosae*	enhancing immune function	
			inhibiting pathogenic bacteria	
27	Mulberry leaves	decrease in *Olsenella* spp.	enhance fermentation	[76]
		decrease in *Megasphaera* spp.		
28	Phytolin-Sugarcane	increase in *Lactobacillus* spp.	improve fermentation	[77]
	polyphenol	increase in *Catenibacterium* spp.	integrity of the gut barrier	
		decrease in *Mohibacterium* spp.	reduced inflammatory response	
		decrease in *Dialister* spp.		
		decrease in *Escherichia-Shigella*		
29	Moutan Cortex Radicis	increase in *Lactobacillus* spp.	improved antioxidant status	[78]
		increase in *Firmicutes*	reduced inflammation	
		decrease in *Bacteroides* spp.		
		decrease in *Parabacteroides* spp.		
		decrease in *Lachnospiraceae* spp.		
		decrease in *Enterococcus* spp.		

**Table 3:** Changes induced by polyphenols in the microbiota and their beneficial effects in mice and humans.

No. crt.	Origin/Type of Polyphenols	Related GI Microbiota	Therapeutic Effect	Ref.
1	Daidzein	* Eggerthellaceae * spp.	lower risk of aortic calcification	[27,89,90,91,92,93,94,95,96]
		increase in *Adlercreutzia equolifaciens*	lower cardiometabolic risk	
		* Adlercreutzia mucosicola *	lower obesity risk	
		* Slackia isoflavoniconvertens *	reduce total serum colesterol	
		* Slackia equolifaciens *	anti-atherogenic effect	
		* Enteroscipio * spp.	hindering carbohydrate metabolism	
		* Lactococcus garvieae *		
		increase in *Bifidobacteriaceae* spp.		
		* Coriobacteriaceae * spp.		
		* Asaccharobacter * spp.		
		* Clostridium * spp.		
		increase in *Bifidobacterium bifidum*		
		decrease in *Bacteroides* spp.		
		decrease in *Faecalibacterium* spp.		
		decrease in *Butyrivibrium* spp.		
		decrease in *Desulfovibrio*		
		increase in *Lactobacillus*		
2	Flavan-3-ols	* Lactiplantibacillus plantarum *	improve vascular function	[27,97,98,99,100,101,102,103,104]
		* Eggerthella lenta *	improve serum cholesterol	
		* Adlercreutzia equolifaciens *	improve glucose metabolism	
		* Flavonifractor plautii *	lower risk of cardiovascular diseases	
		increase in *Lactobacillus* spp.	lower trigricerid concentration	
		increase in *Bifidobacterium* spp.	lower c-reactive protein coincentration	
		increase in *Akkermansia* spp.	lower rsk of Alzeheimer’s diseae	
		decrease in *Clostridium* spp.	lower risk of prostate cancer	
			lower risk of colorectal cancer	
		increase in *Dorea*	improve antioxidant response	
		decrease in *Scillospira*	improve anti-inflammatory response	
		decrease in *Bacteroides*		
3	Flavones/Flavanones	increase in *Bifidobacterium* spp.	lower risk of coronary heart disease	[27,97,105,106,107,108,109,110,111,112]
		* Clostridium * spp.	lower risk of dementia	
		increase in *Bacteroides* spp.	improve vascularization of hippocampal	
		increase in *Lactobacillus* spp.	dental gyrus	
		* Prevotellaceae * spp.	improve memory	
		* Bifidobacterium longum *	decreasing inflammatory cytokines	
		*Eubacterium ramulus*	improve antioxidant response	
		*Clostridium orbiscindens*	improve anti-inflamatory response	
		*Bacteroides distasonis*	lower risk of cancer	
		*Escherichia coli*		
4	Quercitin	increase in *Akkermansia muciniphila*	lower risk of amyotrophic lateral sclerosis	[30]
		decrease in *Enterococcus* spp.		
5	Myricetin	increase in *Actinobacteria* spp.	lower risk of dementia	[113,114]
		increase in *Verrucomicrobia* spp.		
		decrease in *Proteobacteria* spp.		
		decrease in *Bacteroidetes* spp.		
6	Luteolin	increase in *Bifidobacterium* spp.	increase SCFA production	[107]
		increase in *Bacteroides* spp.	lower risk of cancer	
7	Lignans	* Butyribacterium methylotrophicum *	modulate estrogen signaling	[115,116,117,118,119,120,121,122,123]
		* Prevotellaceae callanderi *	modulate lipid metabolism	
		* Prevotellaceae limosum *	decrease the inflamation	
		* PeptoStreptococcus productus *	reduce risk of obesity	
		* Clostridium scindens *	lower risk of cardiovascular diseases	
		* Eggerthella lenta *	lower glucose concentration	
		* Lactobacillus * spp.		
		* Bifidobacterium * spp.		
		increase in *Faecalibacterium prausnitzii*		
		increase in *Alistipes shahii*		
		increase in *Butyrivibrio crossotus*		
		increase in *Methanobrevibacter smithii*		
		increase in *Moryella* spp.		
		increase in *Acetanaerobacterium* spp.		
		increase in *Fastidiosipila* spp.		
		increase in *Streptobacillus* spp.		
8	Ellagic acid/Ellagitannins	increase in *Enterobacteriaceae* spp.	lower risk of cardiovascular diseases	[3,10,20,27,86,124,125,126,127,128,129,130,131,132,133,134,135,136,137,138,139]
		* Gordonibacter pamelaeae *	lower obesity risk	
		* Gordonibacter urolithinfaciens *	lower risk of colorectal cancer	
		* Ellagibacter isourolithinifacien *	improve blood lipid profile	
		* Olsenella * spp.	improve intestinal barier integrity	
		* Senegalimassilia * spp.	decrease the inflamation	
		* Slackia * spp.	lower risk of prostate cancer	
		* Adlercreutzia * spp.	lower risk of breast cancer	
		* Enterocloster bolteae *	lower risk of gastrointestinal diseases	
		* Bifidobacterium pseudocatenulatum *		
		* Lactococcus garvieae *		
		* Enterococcus faecium *		
		* Streptococcus thermophilus *		
		increase in *Akkermansia muciniphila*		
		* Ruminococcaceae *		
		*Lachnospiraceae*		
		decrease in *Clostridium perfringens*		
		decrease in *Methanobrevibacter*		
		decrease in *Escherichia coli*		
9	Resveratrol	increase in *Bacteroidetes* spp.	lower risk of cancer	[30,88,140,141,142,143,144,145,146,147,148]
		* Actinobacteria * spp.	decrease inflamation	
		* Verrucomicrobia * spp.	lower risk of amyotrophic lateral sclerosis	
		* Cyanobacteria * spp.	lower rik of colorectal cancer	
		* Enterobacteriaceae * spp.	lower risk of breast cancer	
		* Slackia equolifaciens *	increase SCFA production	
		* Adlercreutzia equolifaciens *	increase immune response	
		* Bifidobacterium infantis *	increase antioxidant status	
		increase in *Akkermansia muciniphila*	improve serum cholesterol	
		increase in *Butyrivibrio fibrisolvens*	improve glucose metabolism	
		increase in *Lachnospiraceae* spp.	reduce hypertension	
		increase in *Blautia* spp.		
		increase in *LachnoClostridium* spp.		
		increase in *Parabacteroides* spp.		
		increase in *RuminiClostridium* spp.		
		increase in *Bifidobacterium* spp.		
		increase in *Allobaculum*		
		increase in *Ruminococcus*		
		increase in *Clostridium sensu stricto*		
		increase in *Eubacterium*		
		increase in *Ligilactobacillus murinus*		
		decrease in *Psychrobacter*		
		decrease in *Bilophila*		
10	Pterostilbene and Caffeic Acid	increase in *Akkermansia* spp.	increase serotonin concentration	[149,150,151]
		increase in *Odoribacter* spp.	lower risk of amyotrophic lateral sclerosis	
		increase in *Akkermansia muciniphila*	support a healthy lipid profile	
		increase in *Odoribacter splanchnicus*	stimulates the production of butyric acid	
11	Catechin	increase in *Bifidobacterium* spp.	decrease inflamation	
		increase in *Bacteroidetes*	increase SCFA production	[152,153]
		decrease in *Proteobacteria*	lower risk of cancer	
		decrease in *Ruminococcaceae*	raise serum leptin levels	
		decrease in *Lachnospiraceae*		
		increase in *Prevotella*		
		increase in *Parabacteroides*		
		increase in *Fusicatenibacter*		
12	Aronia	increase in *Anaerostipes* spp.	increase SCFA production	[154,155,156]
		increase in *Lawsonibacter asaccharolyticus*	reduced gut-derived endotoxin	
		increase in *Intestinimonas butyriciproducens*	improve antioxidant responseimprove anti-inflammatory response	
		increase in *Lactobacillus*		
		increase in *Ruminococcaceae*		
		increase in *Proteobacteria*		
